# Antibacterial Molecules from Marine Microorganisms against Aquatic Pathogens: A Concise Review

**DOI:** 10.3390/md20040230

**Published:** 2022-03-28

**Authors:** Siya Guo, Zongyi Zhang, Lei Guo

**Affiliations:** 1 Jiangsu Key Laboratory of Marine Bioresources and Environment, Co-Innovation Center of Jiangsu Marine Bio-Industry Technology, Jiangsu Ocean University, Lianyungang 222005, China; 2020220614@jou.edu.cn (S.G.); 2020221253@jou.edu.cn (Z.Z.); 2 Jiangsu Key Laboratory of Marine Biotechnology, School of Food Science and Engineering, Jiangsu Ocean University, Lianyungang 222005, China

**Keywords:** antibacterial molecules, aquatic bacterial pathogens, marine microorganisms

## Abstract

Antibiotic resistance and residues in aquaculture are a growing concern worldwide and consequently identifying favorable antibacterial compounds against aquatic pathogenic bacteria are gained more attention. Active compounds derived from marine microorganisms have shown great promise in this area. This review is aimed to make a comprehensive survey of anti-aquatic pathogenic bacterial compounds that were produced by marine microorganisms. A total of 79 compounds have been reported, covering literature from 1997 to 2021. The compounds are included in different structural classes such as polyketides, terpenoids, nitrogen compounds and others, and some of them present the potential to be developed into agents for the treatment of aquatic pathogenic bacteria.

## 1. Introduction

With the rapid and intensive development of aquaculture, many problems related to aquatic animal diseases and environmental pollution of the water body have gradually been exposed, which has seriously restricted the stable development of aquaculture [1]. The main pathogens of aquatic animals include bacteria, viruses and parasites etc. [2]. It is estimated that China’s annual losses caused by aquatic animal diseases are more than 10 billion yuan. Among them, the economic loss of aquatic animal caused by bacterial diseases accounts for 58%, which is the most serious factor leading to the economic loss of aquaculture [3].

Major bacterial pathogens are *Vibrio*, *Aeromonas*, *Edwardsiella*, *Flavobacterium*, *Pseudomonas*, and *Micrococcus*. *Vibrio* is the most important class of pathogens for bacterial diseases in marine aquaculture. Vibriosis has the characteristics of widespread area and high incidence. The common pathogenic *Vibrio* spp. in aquaculture includes *Vibrio anguillarum*, *Vibrio harveyi*, *Vibrio parahaemolyticus*, and *Vibrio alginolyticus*. *V. anguillarum* is the earliest studied pathogen with high pathogenicity to fish, and the symptom of infection is mainly sepsis. *V. harveyi* is a light-emitting bacterium and a main pathogen of aquatic animals, especially in the nursery and growing stages of shrimp [1]. The main symptoms of *V. harveyi* infection in fish are subcutaneous hemorrhage, redness of the anus, and sides of the skull. *V. parahaemolyticus* can cause inflammation and congestion on the surface of shrimp and marine fish, such as acute hepatopancreatic necrosis disease [4].

*Aeromonas hydrophila* is the main pathogen in the genus *Aeromonas*. Fish infected with *A. hydrophila* are prone to fulminant bleeding disorders, such as erythematosus of carp and loach, and “printing disease” (rotten skin) of catfish [5]. *Edwardsiella tarda* and *Edwardsiella* *ictaluri* are the most common aquatic pathogens in the genus *Edwardsiella*. *E. tarda* and *E. ictaluri* have been found in a variety of farmed freshwater and marine fish, such as eel, flounder, rainbow trout, and catfish. The disease caused by *E. tarda* or *E. ictaluri* is known as enteric septicemia of catfish (ESC) [6]. In addition, yellow mullet, black bass, goldfish, mullet can also be infected.

*Flavobacterium columnare* is a common pathogenic bacterium in the family *Flavobacteriaceae*, and usually attacks the skins, fins and gills of fish. The disease caused by *F. columnare* is often called “columnaris disease” [7]. The symptoms of disease are basically the same, including severe necrosis of gill tissue and skin ulceration from systemic infection. The pathogens of the genus *Pseudomonas* causing fish diseases are mainly *Pseudomonas aeruginosa* and *Pseudomonas fluorescens* etc. *Pseudomonas* can cause diseases in a variety of aquatic animals, such as hemorrhagic, red skin, rot skin, and ulcer diseases [8]. *Micrococcus luteus* is the main cause of hemorrhagic disease in *Monopterus albus*. After infection, the symptoms are diffuse bleeding on the surface, anal swelling and eversion of the anus, and cause high mortality [9].

The antibacterial compounds used in aquaculture are the same used in medicine and veterinary fields. Even though antibiotics are convenient and effective as drugs for the prophylaxis and treatment of bacterial diseases in aquaculture animals, the long-term application or abuse of antibiotics has made antibiotics less and less effective, and mutant pathogens often cause more severe disease. In addition, antibiotic residues in aquatic products directly threaten human health [10]. Therefore, it is urgent to develop new antibacterial agents for aquatic products.

Bio-derived drugs have the advantages of relatively safe, low toxicity, and easy degradation. They are ideal for finding safe and harmless antibacterial raw materials for the aquatic application. At present, marine microorganisms are an important resource for the development of antibacterial agents used in aquaculture. This review article outlines various anti-aquatic pathogenic bacterial molecules produced from marine microorganisms. The availability of these compounds will help develop various applications in the aquaculture field of antibiotics against aquatic bacterial pathogens.

## 2. Marine Bacterial Compounds against Aquatic Pathogenic Bacteria

Marine microbes, especially bacteria and fungi, are excellent producers of natural products with diverse structures and pharmacological activities, and marine microbes serve as valuable resources in the ongoing search for antibacterial compounds against aquatic pathogens [11,12].

**Figure 1:**
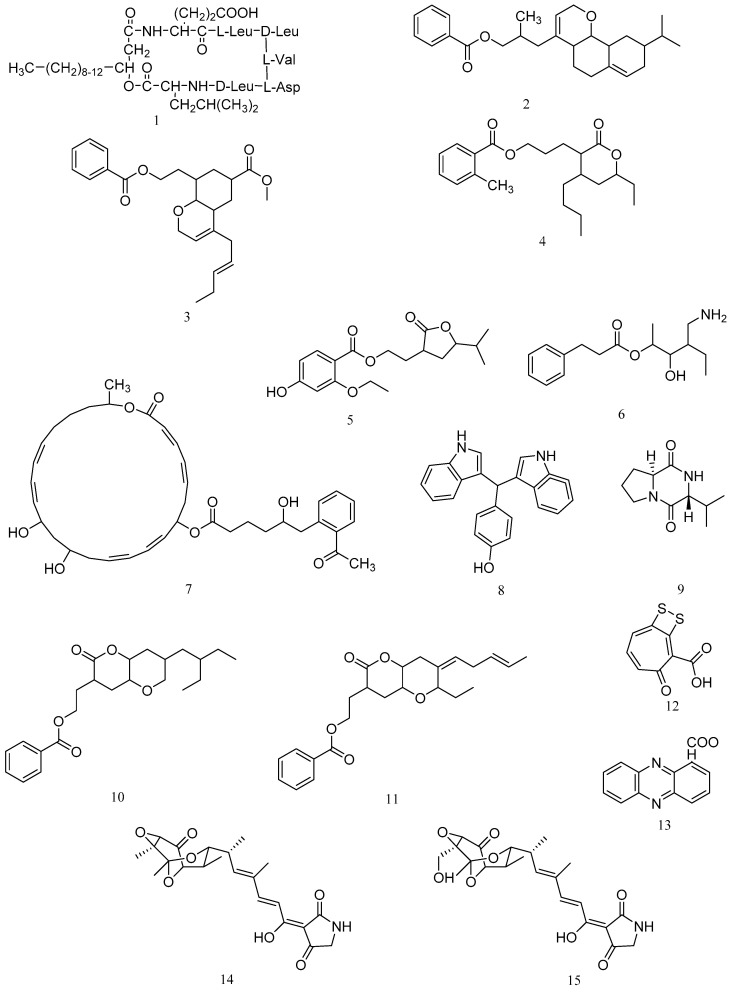
Structures of marine bacterial compounds against aquatic pathogenic bacteria, surfactin (**1**), 3-(octahydro-9-isopropyl-2H-benzo[h]chromen-4-yl)-2-methylpropyl benzoate (**2**), methyl 8-(2-(benzoyloxy)-ethyl)-hexahydro-4-((E)-pent-2-enyl)-2H-chromene-6-carboxylate (**3**), 11-(15-butyl-13-ethyl-tetrahydro-12-oxo-2H-pyran-13-yl) propyl-2-methylbenzoate (**4**), 9-(tetrahydro-12-isopropyl-11-oxofuran-10-yl)-ethyl-4-ethoxy-2-hydroxybenzoate (**5**), 12-(aminomethyl)-11-hydroxyhexanyl-10-phenylpropanoate (**6**), 7-O-6′-(2”-acetylphenyl)-5′-hydroxyhexanoate-macrolactin (**7**), 7,7-bis(3-indolyl)-p-cresol (**8**), cyclo-(S-Pro-R-Val) (**9**), 2-(7-(2-Ethylbutyl)-2,3,4,4a,6,7-hexahydro-2-oxopyrano-[3,2b]-pyran-3-yl)-ethyl benzoate (**10**), 2-((4Z)-2-ethyl-octahydro-6-oxo-3-((E)-pent-3-enylidene)-pyrano-[3,2b]-pyran-7-yl)-ethyl benzoate (**11**), tropodithietic acid (**12**), phenazine-1-carboxylic acid (**13**), tirandamycin A (**14**), and tirandamycin B (**15**).

A cyclic lipopeptide N3 produced by *B. amyloliquefaciens* M1 was identified as surfactin (**1**, Figure 1). The minimal inhibitory concentration (MIC) of the purified lipopeptide N3 against *V. anguillarum* was 1.5 μg/mL (Table 1) [13]. 3-(octahydro-9-isopropyl-2H-benzo[h]chromen-4-yl)-2-methylpropyl benzoate (**2**) and methyl 8-(2-(benzoyloxy)-ethyl)-hexahydro-4-((E)-pent-2-enyl)-2H-chromene-6-carboxylate (**3**) are two polyketides with activity against *Vibrio vulnificus* and were isolated from the ethyl acetate extract of *B. amyloliquefaciens* associated with edible red seaweed, *Laurenciae papillosa*. The compounds **2** and **3** demonstrated significant antibacterial activity against *V. vulnificus* (inhibitory zone diameter of 18.00 ± 1.00 mm and 16.67 ± 0.58 mm, 25 mcg on disk) [14]. Three polyketides from *Bacillus amyloliquefaciens* associated with seaweed *Padina gymnospora* were characterized as 11-(15-butyl-13-ethyl-tetrahydro-12-oxo-2H-pyran-13-yl) propyl-2-methylbenzoate (**4**), 9-(tetrahydro-12-isopropyl-11-oxofuran-10-yl)-ethyl-4-ethoxy-2-hydroxybenzoate (**5**), and 12-(aminomethyl)-11-hydroxyhexanyl-10-phenylpropanoate (**6**). Compounds **4**–**6** displayed significant antibacterial activities against *V. vulnificus* MTCC 1145, *A. hydrophila* MTCC 646, and *V. vulnificus* MTCC 1145 with inhibitory zone diameters of 16.33 ± 0.58 mm, 14.67 ± 1.15 mm, and 17.33 ± 1.00 mm (10 mcg on disk), respectively [15].

Antibacterial aryl-crowned polyketide, 7-O-6′-(2”-acetylphenyl)-5′-hydroxyhexanoate-macrolactin (**7**) was isolated from *Bacillus subtilis* MTCC 10,403 associated with brown seaweed *Anthophycus longifolius*. The MIC assay showed that compound **7** displayed potential antibacterial activities against significant Gram-negative pathogens with MIC of 3.12 μg/mL against *V. vulnificus*, 6.25 μg/mL against *A. hydrophilla*, 12.5 μg/mL against *V. parahaemolyticus* and *P. aeruginosa* [16]. Two compounds including 7,7-bis(3-indolyl)-p-cresol (**8**) and cyclo-(S-Pro-R-Val) (**9**) were isolated from the strain of *Bacillus megaterium* LC derived from the marine sponge *Haliclona oculata*. Compound **8** displayed antibacterial activity at MIC values of 0.05 μg/mL and 0.005 μg/mL against *V. vulnificus* and *M. luteus*. Compound **9** showed antimicrobial activity at MIC value of 0.05 μg/mL against *V. parahaemolyticus* [17].

O-heterocyclic derivatives with antibacterial properties were isolated from *B. subtilis* MTCC 10,407 associated with brown seaweed *Sargassum myriocystum*, and identified as 2-(7-(2-Ethylbutyl)-2,3,4,4a,6,7-hexahydro-2-oxopyrano-[3,2b]-pyran-3-yl)-ethyl benzoate (**10**) and 2-((4Z)-2-ethyl-octahydro-6-oxo-3-((E)-pent-3-enylidene)-pyrano-[3,2b]-pyran-7-yl)-ethyl benzoate (**11**). Compounds **10** and **11** showed significant antibacterial activity (inhibitory zone diameters of 17.66 ± 0.58 mm and 15.3 ± 1.0 mm, 10 μg on disk) against *A. hydrophilla* [18].

An antimicrobial compound produced by *Pseudovibrio* sp. P12, a common and abundant coral-associated bacterium, was identified as tropodithietic acid (**12**), with the MIC value of 0.5 μg/mL against *Vibrio coralliilyticus* and *Vibrio owensii* [19]. A phenazine derivative against *V. anguillarum* was isolated from *Pseudomonas aeruginosa* strain PA31x and demonstrated to be phenazine-1-carboxylic acid (**13**) with the MIC value of 50 μg/mL for *V. anguillarum* [20]. Tirandamycins A (**14**) and B (**15**) were isolated from the crude extract of *Streptomyces tirandamycinicus* sp. nov., a novel marine sponge-derived actinobacterium. Compounds **14** and **15** showed potent antibacterial activity against *Streptococcus agalactiae* with MIC values of 2.52 and 2.55 μg/mL, respectively [21].

## 3. Marine Fungal Compounds against Aquatic Pathogenic Bacteria

### 3.1. Marine Aspergillus

Marine fungi have become the main source of natural products of marine microorganisms due to their complex genetic background, structural diversity and high yields of metabolites. New natural products derived from marine fungi account for about 60% of total marine microbial new natural products and the most studied genera are *Aspergillus* and *Penicillium* [22].

**Figure 2:**
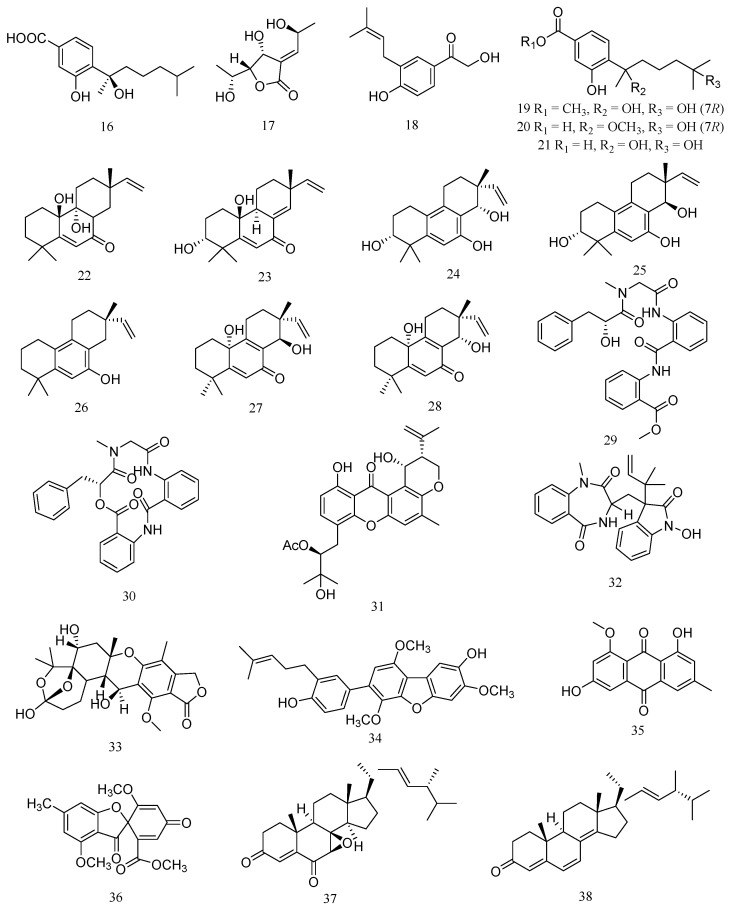
Structures of marine *Aspergillus*-derived compounds against aquatic pathogenic bacteria, (−)-sydonic acid (**16**), asperochrin A (**17**), terreprenphenol A (**18**), ent-aspergoterpenin C (**19**), 7-O-methylhydroxysydonic acid (**20**), hydroxysydonic acid (**21**), aspewentin D (**22**), aspewentin F (**23**), aspewentin G (**24**), aspewentin H (**25**), aspewentin A (**26**), aspewentin I (**27**), aspewentin J (**28**), seco-clavatustide B (**29**), clavatustide B (**30**), aspergixanthone I (**31**), 3-((1-hydroxy-3-(2-methylbut-3-en-2-yl)-2-oxoindolin-3-yl)methyl)-1-methyl-3,4-dihydrobenzo[e][1,4]diazepine-2,5-dione (**32**), austalide R (**33**), 4-methyl-3”-prenylcandidusin A (**34**), questin (**35**), trypacidin (**36**), 7β,8β-epoxy-(22E,24R)-24-methylcholesta-4,22-diene-3,6-dione (**37**), and ergosta-4, 6, 8(14), 22-tetraene-3-one (**38**).

A bisabolane-type sesquiterpenoid, (−)-sydonic acid (**16**), was isolated from marine-derived fungus *Aspergillus* sp. associated with the sponge *Xestospongia testudinaria* (Figure 2). Compound **16** exhibited significant inhibiting activity against *V. Parahaemolyticus* and *V. anguillarum* with MIC values of 10.0 and 5.00 μM (Table 2) [23]. A new polyketide, asperochrin A (**17**), was isolated from *Aspergillus ochraceus* MA-15, which was isolated from the rhizospheric soil of marine mangrove plant *Bruguiera gymnorrhiza*. Compound **17** displayed significant antibacterial activity against *A. hydrophilia*, *V. anguillarum* and *V. harveyi*, with MIC values of 8 μg/mL, 16 μg/mL and 8 μg/mL, respectively [24]. A new prenylated phenol derivative, terreprenphenol A (**18**), was isolated from *Aspergillus terreus* EN-539, which was obtained from the marine red alga *Laurencia okamurai*. Compound **18** displayed potent activity against *A. hydrophila*, *P. aeruginosa*, and *V. harveyi* with MIC values of 2, 2, and 4 μg/mL, respectively [25].

Two new bisabolane-type sesquiterpenoid derivatives, ent-aspergoterpenin C (**19**) and 7-O-methylhydroxysydonic acid (**20**), and a known bisabolane sesquiterpenoid, hydroxysydonic acid (**21**), were isolated from the deep-sea sediment-derived fungus *Aspergillus versicolor* SD-330. Compound **19** exhibited antibacterial activities against *E. tarda*, *P. aeruginosa*, *V. harveyi*, and *V. parahaemolyticus* with MIC value of 8.0 μg/mL. Compound **20** exhibited antibacterial activities against *E. tarda*, *V. anguillarum*, *A. hydrophilia*, *V. harveyi*, and *V. parahaemolyticus* with MIC value of 8.0 μg/mL. Compound **21** exhibited more potent activities against *A. hydrophilia*, *E. tarda*, *V. anguillarum* and *V. harveyi* with MIC value of 4.0 μg/mL [26].

Four new 20-nor-isopimarane diterpenoids, aspewentins D, F, G and H (**22**–**25**), and a known congener, aspewentin A (**26**), were isolated from the deep-sea sediment-derived *Aspergillus wentii* SD-310. Compounds **22**–**26** showed inhibitory activity against the aquatic pathogens *M. luteus*, *E. tarda*, *V. harveyi*, *P. aeruginosa*, and *V. parahemolyticus* with MIC value of 4.0 μg/mL [27]. Meanwhile, two uncommon 20-nor-isopimarane diterpenoid epimers, aspewentin I (**27**) and aspewentin J (**28**) were also isolated from *A. wentii* SD-310. Compounds **27** and **28** showed antibacterial activities against *E. tarda*, *V. harveyi*, and *V. parahaemolyticus* with MIC value of 8.0 μg/mL [28].

Two aminobenzoic peptide, seco-clavatustide B (**29**) and clavatustide B (**30**), were characterized from the Ascidian-derived endophytic fungus *Aspergillus clavatus* AS-107. Compounds **29** exhibited potent activity against *A. hydrophilia*, with a MIC value of 8.2 μM, while compound **30** showed antibacterial activity against *P. aeruginosa*, with a MIC value of 8.8 μM [29]. A new prenylxanthone derivative, aspergixanthone I (**31**), was isolated from the marine-derived fungus *Aspergillus* sp. ZA-01. Compound **31** showed the strongest antibacterial activity against *V. parahemolyticus* (MIC = 1.56 μM), *V. anguillarum* (MIC = 1.56 μM) and *V. alginolyticus* (MIC = 3.12 μM) [30].

A new tryptophan derived alkaloid, 3-((1-hydroxy-3-(2-methylbut-3-en-2-yl)-2-oxoindolin-3-yl)methyl)-1-methyl-3,4-dihydrobenzo[e][1,4]diazepine-2,5-dione (**32**), and a new meroterpenoid, austalide R (**33**), were isolated from the fungus *Aspergillus* sp., isolated from the Mediterranean sponge *Tethya aurantium*. Compound **32** showed significant antibacterial activities against *V. harveyi* and *V. natriegens*, with MIC value of 1 μg/mL. Compound **33** displayed the better potential activity against *V. harveyi* with a MIC value of 0.1 μg/mL [31]. A prenylcandidusin derivative, 4-methyl-3”-prenylcandidusin A (**34**), was isolated from the coral-derived fungus *Aspergillus tritici* SP2-8-1. Compound **34** displayed stronger antibacterial activities against strains of *V. vulnificus*, *V. rotiferianus*, and *V. campbellii*, with MIC values ranging from 7 to 15 μg/mL [32].

Bioassay-guided fractionation resulted in the isolation of an antibacterial compound against *V. harveyi*, questin (**35**), from the marine-derived *Aspergillus* *flavipes* strain HN4-13. Compound **35** exhibited the same anti-*V. harveyi* activity as streptomycin sulfate (MIC 31.25 μg/mL) [1]. Trypacidin (**36**) was isolated from *Aspergillus fumigatus* HX-1 associated with Clams. Compound **36** showed the same anti-*V. harveyi* activity as streptomycin sulfate, with a MIC value of 31.25 µg/mL [33]. 7β,8β-epoxy-(22E,24R)-24-methylcholesta-4,22-diene-3,6-dione (**37**) and ergosta-4, 6, 8(14), 22-tetraene-3-one (**38**) were steroids isolated from the deep sea-derived fungus *Aspergillus penicillioides* SD-311. Compound **37** showed antibacterial activity against *V. anguillarum* with MIC value of 32.0 µg/mL. Compound **38** exhibited inhibitory activity against *E. tarda* and *M. luteus,* with MIC value of 16 μg/mL [34].

### 3.2. Marine Penicillium

**Figure 3:**
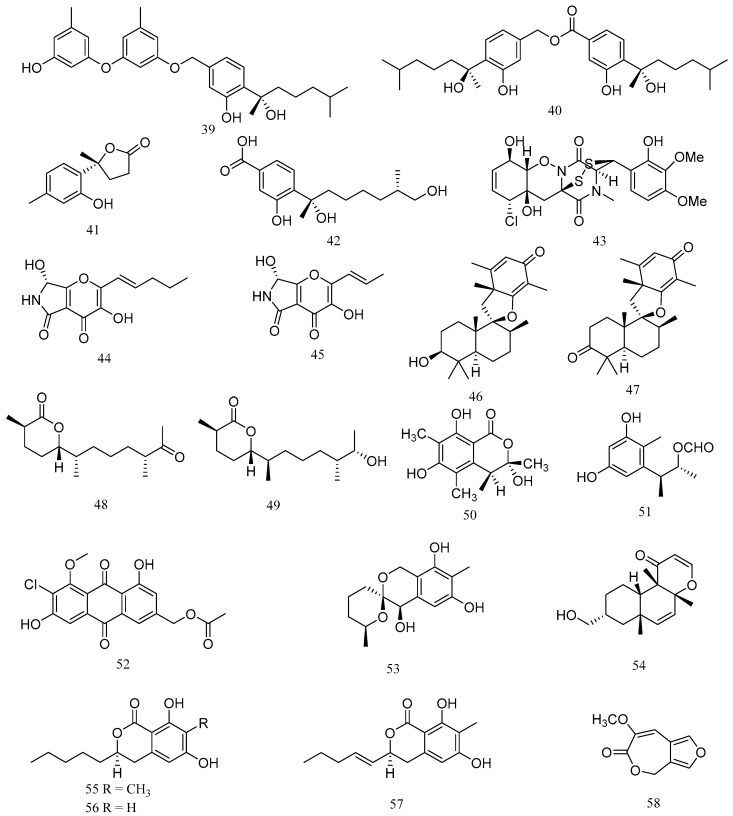
Structures of marine *Penicillium*-derived compounds against aquatic pathogenic bacteria, peniciaculin A (**39**), peniciaculin B (**40**), 1-hydroxyboivinianin A (**41**), (7S,11S)-(+)-12-hydroxysydonic acid (**42**), adametizine A (**43**), pyranonigrin F (**44**), pyranonigrin A (**45**), chermesin A (**46**), chermesin B (**47**), chermesiterpenoid B (**48**), chermesiterpenoid C (**49**), (3S,4S)-sclerotinin A (**50**), citrinin H2 (**51**), 20-acetoxy-7-chlorocitreorosein (**52**), 9-dehydroxysargassopenilline A (**53**), 1,2-didehydropeaurantiogriseol E (**54**), penicisimpin A (**55**), penicisimpin B (**56**), penicisimpin C (**57**), and penicillilactone A (**58**).

Two new phenolic bisabolane sesquiterpenes, peniciaculins A (**39**) and B (**40**), a new nor-bisabolane derivative, 1-hydroxyboivinianin A (**41**), and a known bisabolene, (7S,11S)-(+)-12-hydroxysydonic acid (**42**), were isolated from the deep-sea sediment-derived *Penicillium aculeatum* SD-321 (Figure 3). Compound **39** exhibited antibacterial activity against *V. alginolyticus* with MIC value of 2.0 μg/mL, while compounds **40** and **41** showed inhibitory activity against *E. tarda* and *V. harveyi*, with MIC values of 8.0 and 4.0 μg/mL, respectively. Compound **42** exhibited significant antibacterial activity against *V. parahemolyticus*, with MIC value of 0.5 μg/mL (Table 3) [35].

A new bisthiodiketopiperazine derivative, adametizine A (**43**), was isolated from marine-sponge derived fungus *Penicillium adametzioides* AS-53. Compound **43** showed antibacterial activities against *A. hydrophilia*, *V. harveyi* and *V. parahaemolyticus*, with MIC values of 8, 32, and 8 μg/mL, respectively [36]. A new polyAS-53-dione derivative, pyranonigrin F (**44**), and a related known compound, pyranonigrin A (**45**), were isolated from an endophytic fungus *Penicillium brocae* MA-231, which was obtained from the fresh tissue of the marine mangrove plant *Avicennia marina*. Compounds **44** and **45** displayed significant activity against *V. harveyi* and *V. parahaemolyticus* with MIC values of 0.5 μg/mL [37].

Two new spiromeroterpenoids, chermesins A (**46**) and B (**47**), were isolated from an endophytic fungus *Penicillium chermesinum* EN-480, which was isolated from the inner tissue of the marine red alga *Pterocladiella tenuis*. Compounds **46** and **47** displayed significant activity against *M. luteus*, with MIC value of 8 μg/mL [38]. Meanwhile, two new sesquiterpenoids, chermesiterpenoids B (**48**) and C (**49**), were isolated from *P. chermesinum* EN-480. Compound **48** and **49** exhibited antibacterial activities against *V. anguillarum*, *V. parahaemolyticus* and *M. luteus*, with MIC values of 0.5, 16, and 64 μg/mL, and 1, 32, and 64 μg/mL, respectively [39].

(3S,4S)-sclerotinin A (**50**) and citrinin H2 (**51**) were isolated from the deep sea-derived fungus *Penicillium citrinum* NLG-S01-P1. Compounds **50** and **51** displayed relatively stronger activities against *V. vulnificus* and *V. campbellii*, with MIC values ranging from 15 to 17 μg/mL, respectively [40]. A new chlorinated metabolite, 20-acetoxy-7-chlorocitreorosein (**52**), was isolated from *Penicillium citrinum* HL-5126, an endophytic fungus that was isolated from the mangrove *Bruguiera sexangula var. rhynchopetala* collected in the South China Sea. Compound **52** exhibited antibacterial activity against *V. parahaemolyticus*, with a MIC value of 10 μM [41]. Two new polyketide derivatives, 9-dehydroxysargassopenilline A (**53**) and 1,2-didehydropeaurantiogriseol E (**54**), were isolated from the deep sea-derived fungus *Penicillium cyclopium* SD-413. Compounds **53** and **54** exhibited inhibitory activities against *E. tarda*, *M. luteus*, *V. anguillarum*, and *V. harveyi*, with MIC values ranging from 4 to 32 μg/mL [42].

Three new dihydroisocoumarin derivatives, penicisimpins A–C (**55**–**57**), were isolated from *Penicillium simplicissimum* MA-332, a fungus that was isolated from the rhizosphere of the marine mangrove plant *Bruguiera sexangula var. rhynchopetala*. Compounds **55**–**57** exhibited broad-spectrum inhibitory activities with various MIC values ranging from 4 to >64 mg/mL, with compound **55** showing highest activities against *P. aeruginosa*, *V. parahaemolyticus*, and *V. harveyi*, with MIC value of 4 μg/mL, while compounds **56** and **57** exhibited moderate activities against the tested strains [43]. One novel 7-membered lactone derivative, penicillilactone A (**58**), was isolated from the sponge-derived fungus *Penicillium* sp. LS54. Compound **58** showed antibacterial activity against *V. harveyi*, with a MIC value of 8 μg/mL [44].

### 3.3. Marine Fungi Belonging to Genera Other Than Aspergillus and Penicillium

**Figure 4:**
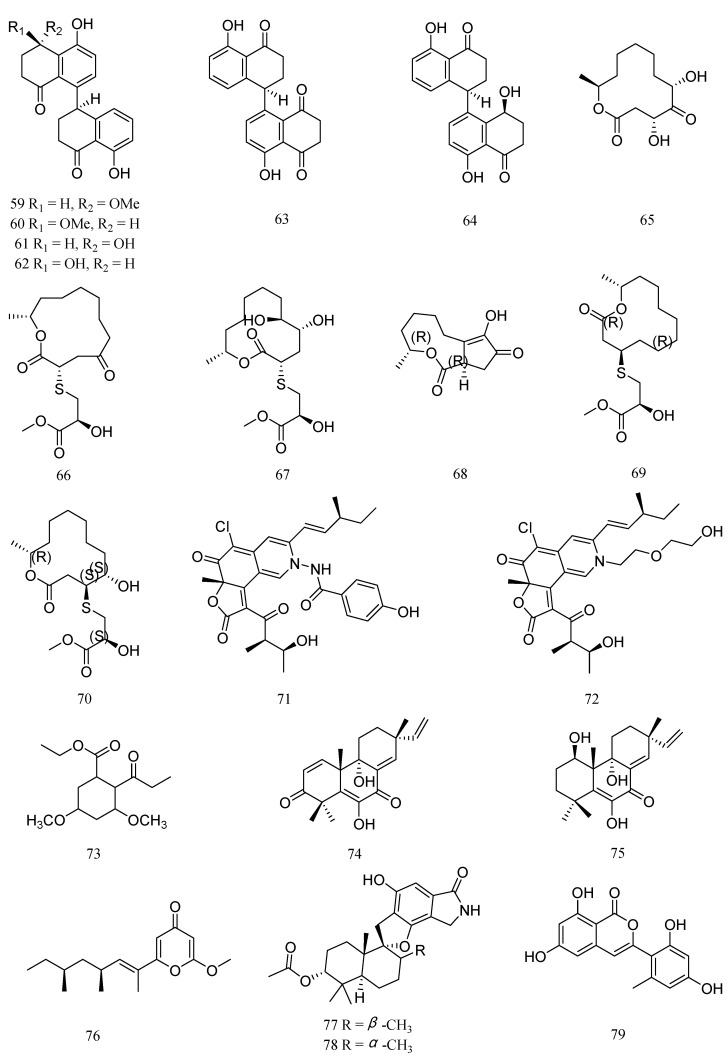
Structures of anti-aquatic pathogenic bacterial compounds isolated from marine fungi belonging to genera other than *Aspergillus* and *Penicillium*, cladosporol F (**59**), cladosporol G (**60**), cladosporol H (**61**), cladosporol I (**62**), cladosporol C (**63**), cladosporol J (**64**), pandangolide 1 (**65**), thiocladospolide A (**66**), thiocladospolide D (**67**), cladocladosin A (**68**), thiocladospolide F (**69**), thiocladospolide G (**70**), chaetoviridide A (**71**), chaetoviridide B (**72**), ethyl 3,5-dimethoxy-2-propionylphenylacetate (**73**), libertellenone M (**74**), libertellenone A (**75**), fusolanone B (**76**), stachybomycin E (**77**), stachybotrylactam acetate (**78**), and trichophenol A (**79**).

Four new cladosporol derivatives, cladosporols F−I (**59**−**62**), the known cladosporol C (**63**), and its new epimer, cladosporol J (**64**) (Figure 4), were isolated from the marine algal-derived endophytic fungus *Cladosporium cladosporioides* EN-399. Compounds **59**−**64** showed inhibitory activities against *M. luteus* and *V. harveyi*, with MIC values of 4−128 μg/mL (Table 4) [45]. Pandangolide 1 (**65**) was isolated from *Cladosporium cladosporioides* MA-299, an endophytic fungus that was isolated from the leaves of the mangrove plant *Bruguiera gymnorrhiza*. Compound **65** exhibited inhibitory activity against *E. ictalurid*, with MIC value of 4.0 μg/mL [46]. Meanwhile, two new 12-membered macrolides, thiocladospolides A (**66**) and D (**67**), were isolated from the same strain, *C. cladosporioides* MA-299. Compounds **66** and **67** exhibited significant activity against *E. tarda* and *E. ictalurid*, with MIC value of 1 μg/mL, respectively [47]. Furthermore, Cladocladosin A (**68**), along with two new sulfur-containing macrolides, thiocladospolides F and G (**69** and **70**), were obtained from *C. cladosporioides* MA-299. Compounds **68**-**70** showed antibacterial activities against *E. tarda* and *V. anguillarum*, with MIC values ranging from 1.0 to 4.0 μg/mL [48].

Two novel compounds, chaetoviridides A (**71**) and B (**72**), were isolated from the deep sea derived fungus *Chaetomium* sp. NA-S01-R1. Compounds **71** and **72** exhibited relatively stronger activities against *V. rotiferianus* and *V. vulnificus*, with MIC values ranging from 7 to 8 μg/mL, respectively [49]. Ethyl 3,5-dimethoxy-2-propionylphenylacetate (**73**) was isolated from the fermentation of *Engyodontium album* derived from deep sea sediment. Compound **73** showed inhibitory activities against *V. vulnificus* with MIC value of 15.6 μg/mL [50]. Two pimarane diterpenes, libertellenones M (**74**) and A (**75**) were isolated from the culture extract of *Eutypella* sp. D-1 derived from high-latitude soil of the Arctic. Compound **74** and **75** displayed inhibitory activity against *V. vulnificus*, with a MIC value of 16 μg/mL [51].

Fusolanone B (**76**) was isolated from a mangrove endophytic fungus *Fusarium solani* HDN15-410. Compound **76** showed inhibitory activity against *V. parahaemolyticus*, with a MIC value of 6.25 μg/mL [52]. A new phenylspirodrimane, stachybomycin E (**77**), and a known compound, stachybotrylactam acetate (**78**), were isolated from the marine-derived fungus *Stachybotrys* sp. SCSIO 40434. Compounds **77** and **78** showed moderate antibacterial activities against *M. luteus* SCSIO ML01, with a MIC value of 8 μg/mL [53]. One new isocoumarin derivative, trichophenol A (**79**), was isolated from *Trichoderma citrinoviride* A-WH-20-3, an endophyte from the marine red alga *Laurencia okamurai*. Compound **79** displayed inhibitory activity against *Pseudoalteromonas citrea*, with a MIC of 16 μg/mL [54].

## 4. Concluding Remarks and Future Prospects

**Figure 5:**
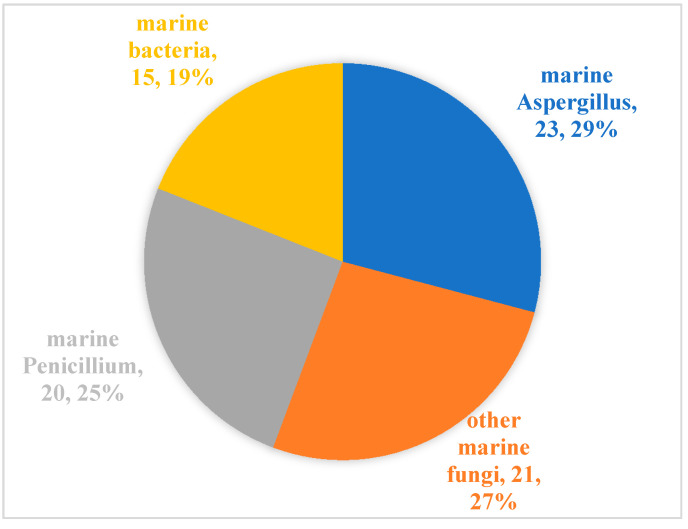
Distribution of marine microorganisms capable of producing active molecules against aquatic pathogenic bacteria.

**Figure 6:**
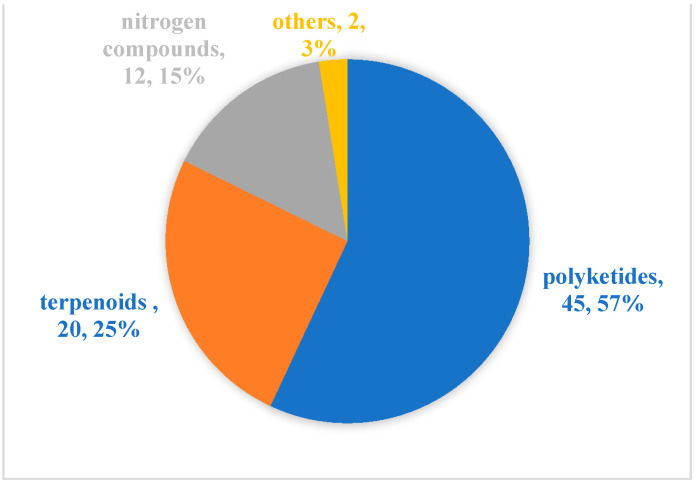
Structural distribution of the anti-aquatic pathogenic bacterial molecules.

Since Cephalosporins were isolated from the secondary metabolites of marine-derived fungus *Acremonium chrysogenum* in 1945, especially since the 1990s, more than 20,000 inspirational natural products with diverse structures and potential bioactivities have been discovered in marine microbes [55]. From our literature review, although marine microbial secondary metabolites have been shown to have diverse biological activities [11,56], there are relatively few reports evaluating their antibacterial activity against aquatic pathogens [13,14,15,16,17,18,19,20,21,22,23,24,25,26,27,28,29,30,31,32,33,34,35,36,37,38,39,40,41,42,43,44,45,46,47,48,49,50,51,52,53,54]. Among the 79 active molecules against aquatic bacterial pathogens, there are only 15 compounds derived from marine bacteria, accounting for 19%. In contrast, antibacterial compounds derived from marine fungi accounted for more than 80%, and were isolated mainly from two genera *Aspergillus* (23, 29%) and *Penicillium* (20, 25%) (Figure 5). When it comes to structural classes, polyketides, terpenoids and nitrogen-containing compounds are the three major structural types of marine microbial-derived anti-aquatic pathogenic bacterial active molecules, accounting for 57%, 25%, and 15%, respectively (Figure 6).

As reported in this review, some marine microbial-derived natural products have good potential against aquatic bacterial pathogens, but there are very few in-depth reports of the in vivo antibacterial efficacy and safety of active molecules [20]. Therefore, further research should focus on the in vivo bacteriostatic effect and safety of marine microbial-derived active compounds against aquatic pathogens in this field.

At present, a limited number of reports on the mechanism of action of anti-aquatic pathogenic active compounds have focused on the changes in bacterial morphology, the inhibition of growth, and the damage in cell membrane and cell wall [13,16,57]. More research is needed to study the mechanism of action of the compounds at the molecular or genetic level.

In conclusion, based on the huge demand for environmentally friendly antibiotic alternatives in the aquaculture industry, further research should deeply evaluate the antibacterial efficacy and safety of marine microbial active molecules in vivo, and investigate their mechanism of action.

## Figures and Tables

**Table 1:** Marine bacterial compounds against aquatic pathogenic bacteria.

Compound	Source Organisms	Activity against	MIC/Zone of Inhibition	References
Surfactin (**1**)	*B. amyloliquefaciens M1*	*V. anguillarum*	1.5 μg/mL	[13]
3-(octahydro-9-isopropyl-2H-benzo[h]chromen-4-yl)-2-methylpropyl benzoate (**2**)	*B. amyloliquefaciens*	*V. vulnificus*	18.00 ± 1.00 mm	[14]
Methyl 8-(2-(benzoyloxy)-ethyl)-hexahydro-4-((E)-pent-2-enyl)-2H-chromene-6-carboxylate (**3**)			16.67 ± 0.58 mm	
11-(15-butyl-13-ethyl-tetrahydro-12-oxo-2H-pyran-13-yl) propyl-2-methylbenzoate (**4**)	*B. amyloliquefaciens*	*V. vulnificus*	16.33 ± 0.58 mm	[15]
9-(tetrahydro-12-isopropyl-11-oxofuran-10-yl)-ethyl-4-ethoxy-2-hydroxybenzoate (**5**)		*A. hydrophila*	14.67 ± 1.15 mm	
12-(aminomethyl)-11-hydroxyhexanyl-10-phenylpropanoate (**6**)		*V. vulnificus*	17.33 ± 1.00 mm	
7-O-6′-(2”-acetylphenyl)-5′-hydroxyhexanoate-macrolactin (**7**)	*B. subtilis* MTCC 10403	*V. vulnificus*	3.12 μg/mL	[16]
		*A. hydrophilla*	6.25 μg/mL	
		*V. parahaemolyticus*	12.5 μg/mL	
		*P. aeruginosa*	12.5 μg/mL	
7,7-bis(3-indolyl)-p-cresol (**8**)	*B. megaterium* LC	*V. vulnificus* *M. luteus*	0.05 μg/mL 0.005 μg/mL	[17]
Cyclo-(S-Pro-R-Val) (**9**)		*V. parahaemolyticus*	0.05 μg/mL	
2-(7-(2-Ethylbutyl)-2,3,4,4a,6,7-hexahydro-2-oxopyrano-[3,2b]-pyran-3-yl)-ethyl benzoate (**10**)	*B. subtilis* MTCC 10407	*A. hydrophilla*	17.66 ± 0.58 mm	[18]
2-((4Z)-2-ethyl-octahydro-6-oxo-3-((E)-pent-3-enylidene)-pyrano-[3,2b]-pyran-7-yl)-ethyl benzoate (**11**)			15.3 ± 1.0 mm	
Tropodithietic acid (**12**)	*Pseudovibrio* sp. P12	*V. coralliilyticus* *V. owensii*	0.5 μg/mL	[19]
Phenazine-1-carboxylic acid (**13**)	*P. aeruginosa* PA31x	*V. anguillarum*	50 μg/mL	[20]
Tirandamycin A (**14**)	*S. tirandamycinicus* sp. nov.	*Streptococcus agalactiae*	2.52 μg/mL	[21]
Tirandamycin B (**15**)			2.55 μg/mL	

**Table 2:** Marine *Aspergillus*-derived compounds against aquatic pathogenic bacteria.

Compound	Source Organisms	Activity against	MIC	References
(−)-sydonic acid (**16**)	*Aspergillus* sp.	*V. Parahaemolyticus*	10.0 μM	[23]
		*V. anguillarum*	5.00 μM	
Asperochrin A (**17**)	*A. ochraceus* MA-15	*A. hydrophilia*	8 μg/mL	[24]
		*V. anguillarum*	16 μg/mL	
		*V. harveyi*	8 μg/mL	
Terreprenphenol A (**18**)	*A. terreus* EN-539	*A. hydrophila*	2 μg/mL	[25]
		*P. aeruginosa*	2 μg/mL	
		*V. harveyi*	4 μg/mL	
Ent-aspergoterpenin C (**19**)	*A. versicolor* SD-330	*E. tarda*	8 μg/mL	[26]
		*P. aeruginosa*	8 μg/mL	
		*V. harveyi*	8 μg/mL	
		*V. parahaemolyticus*	8 μg/mL	
7-O-methylhydroxysydonic acid (**20**)		*E. tarda*	4 μg/mL	
		*V. anguillarum*	4 μg/mL	
		*A. hydrophilia*	8 μg/mL	
		*V. harveyi*	8 μg/mL	
		*V. parahaemolyticus*	8 μg/mL	
Hydroxysydonic acid (**21**)		*A. hydrophilia*	4 μg/mL	
		*E. tarda*	4 μg/mL	
		*V. anguillarum*	4 μg/mL	
		*V. harveyi*	4 μg/mL	
Aspewentin D (**22**)	*A. wentii* SD-310	*M. luteus*	4 μg/mL	[27,28]
Aspewentin F (**23**)		*E. tarda*	4 μg/mL	
		*V. harveyi*	4 μg/mL	
Aspewentin G (**24**)		*V. harveyi*	4 μg/mL	
Aspewentin H (**25**)		*P. aeruginosa*	4 μg/mL	
Aspewentin A (**26**)		*V. parahaemolyticus*	4 μg/mL	
Aspewentin I (**27**)		*E. tarda*	8 μg/mL	
		*V. harveyi*	8 μg/mL	
		*V. parahaemolyticus*	8 μg/mL	
Aspewentin J (**28**)		*E. tarda*	8 μg/mL	
		*V. harveyi*	8 μg/mL	
		*V. parahaemolyticus*	8 μg/mL	
Seco-clavatustide B (**29**)	*A. clavatus* AS-107	*A. hydrophilia*	8.2 μM	[29]
Clavatustide B (**30**)		*P. aeruginosa*	8.8 μM	
Aspergixanthone I (**31**)	*Aspergillus* sp. ZA-01	*V. parahemolyticus*	1.56 μM	[30]
		*V. anguillarum*	1.56 μM	
		*V. alginolyticus*	3.12 μM	
3-((1-hydroxy-3-(2-methylbut-3-en-2-yl)-2-oxoindolin-3-yl)methyl)-1-methyl-3,4-dihydrobenzo[e][1,4]diazepine-2,5-dione (**32**)	*Aspergillus* sp.	*V. harveyi*	1 μg/mL	[31]
		*V. natriegens*	1 μg/mL	
Austalide R (**33**)	*Aspergillus* sp.	*V. harveyi*	0.1 μg/mL	
4-methyl-3”-prenylcandidusin A (**34**)	*A. tritici* SP2-8-1	*V. vulnificus*	7.77 μg/mL	[32]
		*V. rotiferianus*	7.75 μg/mL	
		*V. campbellii*	15.6 μg/mL	
Questin (**35**)	*A. flavipes* HN4-13	*V. harveyi*	31.25 μg/mL	[1]
Trypacidin (**36**)	*A. fumigatus* HX-1	*V. harveyi*	31.25 µg/mL	[33]
7β,8β-epoxy-(22E,24R)-24-methylcholesta-4,22-diene-3,6-dione (**37**)	*A. penicillioides* SD-311	*V. anguillarum*	32.0 µg/mL	[34]
ergosta-4, 6, 8(14), 22-tetraene-3-one (**38**)		*E. tarda*	16.0 µg/mL	
		*M. luteus*		

**Table 3:** Marine *Penicillium*-derived compounds against aquatic pathogenic bacteria.

Compound	Source Organisms	Activity against	MIC	References
Peniciaculin A (**39**)	*P. aculeatum* SD-321	*V. alginolyticus*	2.0 μg/mL	[35]
Peniciaculin B (**40**)		*E. tarda*	8.0 μg/mL	
1-hydroxyboivinianin A (**41**)		*V. harveyi*	4.0 μg/mL	
(7S,11S)-(+)-12-hydroxysydonic acid (**42**)		*V. parahemolyticus*	0.5 μg/mL	
Adametizine A (**43**)	*P. adametzioides* AS-53	*A. hydrophilia*	8 μg/mL	[36]
		*V. harveyi*	32 μg/mL	
		*V. parahaemolyticus*	8 μg/mL	
Pyranonigrin F (**44**)	*P. brocae* MA-231	*V. harveyi*	0.5 μg/mL	[37]
		*V. parahaemolyticus*	0.5 μg/mL	
Pyranonigrin A (**45**)		*V. harveyi*	0.5 μg/mL	
		*V. parahaemolyticus*	0.5 μg/mL	
Chermesin A (**46**)	*P. chermesinum* EN-480	*Micrococcus luteus*	8 μg/mL	[38]
Chermesin B (**47**)		*Micrococcus luteus*	8 μg/mL	
Chermesiterpenoid B (**48**)		*V. anguillarum*	0.5 μg/mL	[39]
		*V. parahaemolyticus*	16 μg/mL	
		*M. luteus*	64 μg/mL	
Chermesiterpenoid C (**49**)		*V. anguillarum*	1 μg/mL	
		*V. parahaemolyticus*	32 μg/mL	
		*M. luteus*	64 μg/mL	
(3S,4S)-sclerotinin A (**50**)	*P. citrinum* NLG-S01-P1	*V. vulnificus*	16.6 μg/mL	[40]
		*V. campbellii*	15.3 μg/mL	
Citrinin H2 (**51**)		*V. vulnificus*	15.7 μg/mL	
		*V. campbellii*	15.6 μg/mL	
20-acetoxy-7-chlorocitreorosein (**52**)	*P. citrinum* HL-5126	*V. parahaemolyticus*	10 μM	[41]
9-dehydroxysargassopenilline A (**53**)	*P. cyclopium* SD-413	*E. tarda*	16 μg/mL	[42]
		*M. luteus*	4 μg/mL	
		*V. anguillarum*	32 μg/mL	
1,2-didehydropeaurantiogriseol E (**54**)		*M. luteus*	32 μg/mL	
		*V. harveyi*	4 μg/mL	
		*V. anguillarum*	4 μg/mL	
Penicisimpin A (**55**)	*P. simplicissimum* MA-332	*P. aeruginosa*	4 μg/mL	[43]
		*V. parahaemolyticus*	4 μg/mL	
		*V. harveyi*	4 μg/mL	
		*M. luteus*	8 μg/mL	
		*V. alginolyticus*	8 μg/mL	
Penicisimpin B (**56**)		*P. aeruginosa*	32 μg/mL	
		*V. parahaemolyticus*	32 μg/mL	
		*V. harveyi*	16 μg/mL	
		*M. luteus*	64 μg/mL	
		*V. alginolyticus*	32 μg/mL	
		*A. hydrophilia*	32 μg/mL	
Penicisimpin C (**57**)		*P. aeruginosa*	8 μg/mL	
		*V. parahaemolyticus*	8 μg/mL	
		*V. harveyi*	8 μg/mL	
		*M. luteus*	16 μg/mL	
		*V. alginolyticus*	16 μg/mL	
		*A. hydrophilia*	16 μg/mL	
Penicillilactone A (**58**)	*Penicillium* sp. LS54	*V. harveyi*	8 μg/mL	[44]

**Table 4:** Anti-aquatic pathogenic bacterial compounds isolated from marine fungi belonging to genera other than *Aspergillus* and *Penicillium*.

Compounds	Source Organisms	Activity against	MIC	References
Cladosporol F (**59**)	*C. cladosporioides* EN-399	*M. luteus*	64 μg/mL	[45]
		*V. harveyi*	32 μg/mL	
Cladosporol G (**60**)		*M. luteus*	128 μg/mL	
		*V. harveyi*	64 μg/mL	
Cladosporol H (**61**)		*M. luteus*	64 μg/mL	
		*V. harveyi*	4 μg/mL	
Cladosporol I (**62**)		*M. luteus*	64 μg/mL	
		*V. harveyi*	16 μg/mL	
Cladosporol C (**63**)		*M. luteus*	32 μg/mL	
		*V. harveyi*	16 μg/mL	
Cladosporol J (**64**)		*M. luteus*	64 μg/mL	
		*V. harveyi*	32 μg/mL	
Pandangolide 1 (**65**)	*C. cladosporioides* MA-299	*E. ictaluri*	4 μg/mL	[46]
Thiocladospolide A (**66**)		*E. tarda*	1 μg/mL	[47]
Thiocladospolide D (**67**)		*E. ictaluri*	1 μg/mL	
Cladocladosin A (**68**)		*E. tarda*	1 μg/mL	[48]
		*P. aeruginosa*	4 μg/mL	
		*V. anguillarum*	2 μg/mL	
Thiocladospolide F (**69**)		*E. tarda*	2 μg/mL	
		*V. anguillarum*	2 μg/mL	
Thiocladospolide G (**70**)		*E. tarda*	2 μg/mL	
		*V. anguillarum*	4 μg/mL	
Chaetoviridide A (**71**)	*Chaetomium* sp. NA-S01-R1	*V. rotiferianus*	7.3 μg/mL	[49]
Chaetoviridide B (**72**)		*V. vulnificus*	7.4 μg/mL	
Ethyl 3,5-dimethoxy-2-propionylphenylacetate (**73**)	*Engyodontium album*	*V. vulnificus*	15.6 μg/mL	[50]
Libertellenone M (**74**)	*Eutypella* sp. D-1	*V. vulnificus*	16 μg/mL	[51]
Libertellenone A (**75**)		*V. vulnificus*	16 μg/mL	
Fusolanone B (**76**)	*Fusarium solani* HDN15-410	*V. parahaemolyticus*	6.25 μg/mL	[52]
Stachybomycin E (**77**)	*Stachybotrys sp. SCSIO 40434*	*M. luteus*	8 μg/mL	[53]
Stachybotrylactam acetate (**78**)		*M. luteus*	8 μg/mL	
Trichophenol A (**79**)	*Trichoderma citrinoviride* A-WH-20-3	*Pseudoalteromonas citrea*	16 μg/mL	[54]
